# Discovery and validation of GNA12^circle^ as a first-trimester plasma eccDNA marker for early-onset preeclampsia

**DOI:** 10.3389/fneur.2026.1900036

**Published:** 2026-08-25

**Authors:** Jin Wang, Fei Hou, Yingying Peng, Juntian Hao, Hua Jin

**Affiliations:** 1 Prenatal Diagnosis Center, Jinan Maternity and Child Care Hospital Affiliated to Shandong First Medical University, Jinan, Shandong, China; 2 Graduate School, Shandong First Medical University, Jinan, Shandong, China

**Keywords:** circulating biomarker, early-onset preeclampsia, extrachromosomal circular DNA, risk prediction, vascular risk

## Abstract

**Background:**

Early-onset preeclampsia (EOPE) is a major cause of maternal and perinatal morbidity and is characterized by placental dysfunction, systemic endothelial injury, and hypertensive vascular stress. Because hypertensive disorders of pregnancy may also signal later maternal cardiovascular and cerebrovascular vulnerability, effective biomarkers for first-trimester risk assessment remain clinically important. Extrachromosomal circular DNA (eccDNA), a stable form of circulating cell-free DNA, has emerged as a potential source of disease-associated biomarkers. This study aimed to characterize first-trimester plasma eccDNA alterations associated with subsequent EOPE and to identify and validate a candidate circulating eccDNA marker for early risk assessment.

**Methods:**

A two-stage nested case–control study was conducted within a prospective birth cohort. In the discovery stage, plasma samples collected at 11–13 weeks of gestation from 5 women who subsequently developed EOPE and 5 matched normotensive controls were profiled by Circle-Seq to characterize genome-wide eccDNA alterations. Candidate eccDNAs were prioritized through differential abundance analysis and were further confirmed by outward PCR and Sanger sequencing. In the validation stage, the candidate selected marker was quantified by junction-specific qPCR in an independent cohort of 109 EOPE cases and 109 controls. Its potential predictive value was further evaluated alone and in combination with routine first-trimester clinical variables.

**Results:**

In the exploratory discovery analysis, 410 nominally differentially abundant candidate eccDNAs were identified as a hypothesis-generating pool. Among these, GNA12^circle^ (chr7:2876332–2,876,692) was prioritized and experimentally validated at the circular junction. In the independent validation cohort, plasma GNA12^circle^ levels were significantly higher in women who later developed EOPE than in controls. When combined with routine first-trimester variables, GNA12^circle^ improved predictive performance. The RF model showed the best overall cross-validated performance among the evaluated classifiers, with a mean held-out test-fold AUC of 0.843.

**Conclusion:**

First-trimester plasma eccDNA profiling revealed distinct alterations associated with subsequent EOPE, from which GNA12^circle^ was identified and validated as a candidate circulating marker. These findings support further investigation of circulating eccDNA for early EOPE risk assessment in larger multicenter populations.

## Introduction

Preeclampsia (PE) is a pregnancy-specific hypertensive disorder that affects approximately 2–8% of pregnancies worldwide (1, 2). As a multisystem disorder, PE may progress to severe maternal morbidity involving renal, hepatic, neurologic and hematologic complications, and is also linked to adverse perinatal outcomes, including fetal growth restriction and medically indicated preterm birth (2–4). PE is widely considered a placental disorder characterized by placental dysfunction and maternal endothelial impairment (3). Because delivery of the fetus and placenta is the only definitive treatment, effective management depends on early risk stratification so that prevention and surveillance can begin in time (1, 2). Notably, prophylactic low-dose aspirin initiated before 16 weeks’ gestation has been shown to reduce the risk of preterm and early-onset preeclampsia (EOPE) among high-risk women (1, 5–7). In addition, prenatal physical activity and Mediterranean-style dietary patterns are linked to lower PE risk, adding to the case for identifying at-risk pregnancies before symptoms appear (8, 9).

Over the past decade, considerable effort has focused on developing first-trimester screening strategies for PE (10). Current best-practice approaches combine maternal demographic risk factors with biophysical and biochemical markers to estimate an individual’s risk. Adding placental biomarkers, particularly placental growth factor (PlGF), to clinical factors and uterine artery Doppler assessment can substantially improve early prediction of PE (3, 11). For instance, the Fetal Medicine Foundation (FMF) algorithm uses basic maternal information, mean arterial pressure, uterine artery pulsatility index, and circulating markers such as PAPP-A and PlGF to identify high-risk pregnant women, enabling targeted prophylaxis (12). These multimodal models have achieved high detection rates for PE in research settings. Nevertheless, a substantial proportion of EOPE still arise outside conventional high-risk groups, which leaves room for new biomarkers and complementary strategies to sharpen early risk stratification.

One emerging frontier is the analysis of circulating nucleic acids. Cell-free DNA has transformed prenatal screening for fetal aneuploidies, and related plasma-based approaches are now being extended to obstetric complications (13, 14). Extrachromosomal circular DNA (eccDNA) is a structurally distinct class of cell-free DNA that exists as covalently closed circles in human tissues and plasma and may offer complementary insight into placental pathophysiology. Because of their circular configuration, eccDNAs are relatively resistant to exonuclease degradation and may exhibit enhanced stability in the circulation (15, 16). High-throughput approaches such as Circle-Seq enable unbiased profiling of eccDNA without pre-specifying genomic targets, facilitating discovery of disease-associated signatures (17). Beyond oncology, eccDNA has been investigated as a potential biomarker in an expanding range of conditions, including metabolic and immune-mediated diseases, and emerging pregnancy studies suggest that eccDNA profiles may reflect placental dysfunction and complications such as gestational diabetes mellitus and fetal growth restriction (18–22).

Despite growing interest in eccDNA, evidence linking first-trimester plasma eccDNA to pre-eclampsia prediction remains limited. Maternal plasma contains both maternal- and fetal-derived eccDNA; fetal-origin eccDNAs tend to be shorter and are rapidly cleared after delivery, supporting a placental contribution to circulating eccDNA dynamics (23, 24). Mechanistic studies further suggest that eccDNA may accumulate in preeclamptic placentas and engage inflammatory or cell-death pathways in trophoblasts (25). Nevertheless, no eccDNA-based biomarker has yet been established for early prediction of PE, and the clinical utility of measuring plasma eccDNA at the routine 11–13 week visit for reproducible risk stratification remains to be defined.

In this study, we evaluated first-trimester plasma eccDNA as a candidate biomarker class for risk stratification of EOPE using a discovery-to-validation design. Maternal plasma collected at 11–13 weeks’ gestation underwent unbiased eccDNA profiling, followed by orthogonal confirmation and targeted quantification of a prioritized eccDNA marker. We then examined whether incorporating the eccDNA signal alongside routinely available first-trimester variables (including PAPP-A MoM, beta-hCG MoM, NT MoM and maternal characteristics) could inform prediction models for subsequent EOPE. By integrating a targeted molecular readout with routinely available first-trimester variables, this work aimed to evaluate whether plasma eccDNA could provide complementary information for early EOPE risk assessment.

## Methods

### Study design and sample collection

This study was designed as a two-stage case–control investigation nested within a prospective birth cohort in Jinan, China. We recruited singleton pregnant women at 11–13 weeks of gestation from Jinan Maternity and Child Care Hospital Affiliated to Shandong First Medical University. Women were excluded if they had pre-existing conditions such as chronic hypertension, diabetes, kidney or autoimmune diseases, multiple pregnancies, or confirmed fetal chromosomal abnormalities or major structural defects. From this cohort, women who later developed EOPE and matched controls who remained normotensive throughout pregnancy were identified. Participants were followed up until delivery to ascertain pregnancy outcomes. EOPE was diagnosed according to international criteria as new-onset hypertension (systolic blood pressure ≥140 mmHg and/or diastolic blood pressure ≥90 mmHg) arising after 20 weeks’ gestation, together with either proteinuria (≥300 mg/24 h or equivalent) or, in the absence of proteinuria, maternal organ dysfunction (26, 27). EOPE was defined as preeclampsia diagnosed before 34 weeks’ gestation. The study protocol was approved by the Ethics Committee of Jinan Maternity and Child Care Hospital (IRB No. KY-23-37), and written informed consent was obtained from all participants in accordance with the Declaration of Helsinki.

In total, 114 women who subsequently developed EOPE and 114 normotensive controls in a 1: 1 ratio were included from this cohort. First-trimester plasma samples were collected from all participants at 11–13 weeks’ gestation. The study consisted of two non-overlapping phases: a discovery phase and an independent validation phase. For unbiased eccDNA profiling, a discovery subset of 5 EOPE cases and 5 matched controls was selected. Importantly, these 10 participants were excluded from all downstream targeted validation and model development to avoid participant overlap and potential information leakage. The validation cohort therefore comprised the remaining 109 EOPE cases and 109 matched controls, in whom targeted eccDNA quantification and prediction modeling were performed. The clinical characteristics of the EOPE and control groups are summarized in Table 1.

**Table 1:** Baseline clinical characteristics in early-onset preeclampsia and control groups.

Variables	Stat	All	Control	EOPE	*p*-value
Maternal age	*n*	218	109	109	0.198
	Mean ± SD	28.53 ± 3.39	28.83 ± 3.40	28.23 ± 3.37	
	M (Q1, Q3)	29.00 (26.00, 31.00)	29.00 (26.00, 32.00)	28.00 (25.00, 31.00)	
	Min, Max	20.00,38.00	21.00,38.00	20.00,34.00	
Height	*n*	218	109	109	0.696
	Mean ± SD	162.00 ± 4.96	161.91 ± 5.04	162.09 ± 4.91	
	M (Q1, Q3)	162.00 (158.00, 165.00)	162.00 (158.00, 165.00)	162.00 (159.00, 165.00)	
	Min, Max	148.00,175.00	150.00,174.00	148.00,175.00	
Weight	*n*	218	109	109	0.003
	Mean ± SD	63.73 ± 11.74	61.45 ± 10.58	66.01 ± 12.43	
	M (Q1, Q3)	62.00 (55.00, 70.88)	58.00 (54.00, 66.60)	65.00 (55.50, 73.00)	
	Min, Max	45.00,105.00	45.00,105.00	45.00,102.00	
Maternal BMI	*n*	218	109	109	0.004
	Mean ± SD	24.32 ± 4.55	23.48 ± 4.14	25.16 ± 4.80	
	M (Q1, Q3)	23.49 (21.09, 27.06)	22.66 (20.78, 25.14)	24.57 (21.48, 28.12)	
	Min, Max	15.92,42.06	15.92,42.06	16.94,38.87	
Gestational age at sampling	*n*	218	109	109	0.046
	Mean ± SD	12.28 ± 0.59	12.38 ± 0.65	12.19 ± 0.52	
	M (Q1, Q3)	12.30 (12.00, 12.60)	12.30 (12.10, 13.00)	12.20 (11.60, 12.50)	
	Min, Max	11.10,13.60	11.10,13.60	11.20,13.40	
CRL	*n*	218	109	109	0.047
	Mean ± SD	59.75 ± 7.75	60.94 ± 8.63	58.55 ± 6.59	
	M (Q1, Q3)	59.00 (54.00, 65.00)	60.00 (55.00, 68.00)	57.00 (53.00, 64.00)	
	Min, Max	44.00,79.00	44.00,79.00	45.00,76.00	
NT	*n*	218	109	109	0.101
	Mean ± SD	1.03 ± 0.22	1.05 ± 0.22	1.00 ± 0.21	
	M (Q1, Q3)	1.02 (0.89, 1.15)	1.06 (0.90, 1.17)	1.00 (0.86, 1.14)	
	Min, Max	0.50,1.84	0.58,1.84	0.50,1.70	
PAPPA MoM	*n*	218	109	109	0.005
	Mean ± SD	1.07 ± 0.74	1.20 ± 0.78	0.94 ± 0.68	
	M (Q1, Q3)	0.94 (0.61, 1.36)	1.03 (0.71, 1.50)	0.88 (0.50, 1.21)	
	Min, Max	0.09,5.79	0.17,5.79	0.09,5.69	
beta-hCG MoM	*n*	218	109	109	0.738
	Mean ± SD	1.36 ± 0.90	1.31 ± 0.70	1.41 ± 1.06	
	M (Q1, Q3)	1.17 (0.78, 1.70)	1.09 (0.86, 1.68)	1.19 (0.70, 1.72)	
	Min, Max	0.28,6.53	0.30,3.50	0.28,6.53	

Data are presented as mean ± standard deviation (SD), median (Q1, Q3), or minimum and maximum values, as appropriate. p-values were calculated using Student’s t-test or Mann–Whitney U test for continuous variables, depending on distribution. BMI, body mass index; CRL, crown–rump length; NT, nuchal translucency; MoM, multiples of the median; EOPE, early-onset preeclampsia.

### Sample collection and processing

At 11–13 weeks’ gestation, approximately 10 mL of maternal peripheral blood was collected from each participant into EDTA anticoagulant tubes. The blood was centrifuged at 1,600 × g for 10 min at 4 °C to separate the plasma. The plasma was carefully transferred to Eppendorf (EP) tubes and was centrifuged again at 16,000 × g for 10 min at 4 °C to remove any residual cells and debris. The resulting cell-free plasma supernatant was aliquoted and stored at −80 °C until DNA extraction.

### EccDNA extraction, library preparation, and circle-Seq sequencing

EccDNA was sequenced using a high-throughput platform by CloudSeq Biotech Inc. (Shanghai, China), with procedures adapted from published methods (28). Circle-Seq was applied to identify eccDNA in 5 EOPE cases and 5 controls. Cell-free DNA was extracted from 2 mL of plasma, following the manufacturer’s protocol. To digest linear double-stranded DNA, extracted DNA was treated with Plasmid-Safe ATP-dependent DNase in the presence of ATP. Then, the reaction was heat-inactivated, and the DNA was purified and concentrated using a column-based cleanup kit (MinElute, Qiagen).

Sequencing libraries were prepared using the Illumina Nextera XT DNA Library Prep Kit (Illumina, United States) with minor adjustments for circular DNA. Circular molecules were fragmented and tagged with adapters by Nextera transposase tagmentation. Library preparation followed the manufacturer’s instructions, unique dual indices were added for multiplexing, and libraries were PCR-amplified, quantified, and assessed for size distribution using an Agilent Bioanalyzer. The 10 libraries were pooled in equimolar amounts and sequenced on an Illumina NovaSeq 6,000 platform to generate 150 bp paired-end reads.

### Bioinformatics identification of eccDNA and differential analysis

Sequencing was performed on an Illumina NovaSeq 6,000 with >90% bases at Q30 (~99.9% accuracy). Adapters and low-quality bases were removed with Cutadapt, and clean reads were aligned to the hg19/GRCh37 human reference genome using BWA-MEM; all genomic coordinates refer to hg19. Aligned reads were analyzed with Circle-Map in split-read mode to detect eccDNAs, and candidates supported by at least two reads spanning the junction and not overlapping artifacts or low-complexity regions were retained. eccDNA abundance was quantified as soft-clipped or chimeric reads at each junction using Samtools, assembled into a count matrix for the 10 discovery samples, and scaled by total read number. Because eccDNA identification and quantification rely primarily on junction-spanning reads, eccDNA count matrices are typically sparse. For differential screening, eccDNA counts between EOPE and control samples were compared using edgeR. FDR values were calculated for multiple-testing adjustment and reported for transparency. However, given the sparse junction-read-based nature of eccDNA data, candidate eccDNAs were initially screened for exploratory validation using fold-change and nominal *p*-value thresholds, defined as fold change ≥2.0 or ≤0.5 and nominal *p* value <0.05. No candidate eccDNA reached FDR < 0.05 after correction; therefore, these candidates were interpreted as nominally differentially abundant exploratory signals rather than genome-wide significant findings. Prioritized candidates were subsequently evaluated by orthogonal validation, including outward PCR, Sanger sequencing, and junction-specific qPCR. Genomic annotation was performed with BEDTools, classifying eccDNAs by genomic region and size distribution.

Candidate eccDNAs for experimental validation were nominated from the exploratory candidate pool using a stepwise prioritization framework. Candidate eccDNAs were first screened based on nominal differential abundance between EOPE and control samples. Technical feasibility was then assessed according to fragment length suitable for outward PCR/Sanger sequencing and junction-specific qPCR, feasibility of designing specific primers around the predicted circular junction, and detection patterns across discovery samples. Biological plausibility of the corresponding genomic locus was considered after the initial statistical screening and before targeted validation, based on available evidence related to EOPE, placental dysfunction, trophoblast biology, endothelial dysfunction, or vascular signaling. This prioritization process was exploratory and was not intended to represent a formal genome-wide ranking algorithm based solely on logFC, *p* value, or FDR. To improve transparency, the top 10 increased-abundance and top 10 decreased-abundance candidate eccDNAs prioritized under this framework are summarized in Supplementary Table S1.

### Experimental validation of GNA12^circle^ by outward PCR and sanger sequencing

Outward-facing PCR was used to validate the candidate eccDNA GNA12^circle^. Primers were designed from the Circle-Seq junction coordinates (Chr7:2876332–2,876,692, hg19) using NCBI Primer-BLAST. Primers were designed to anneal to regions flanking the predicted junction on each side and were oriented outward so that an amplicon would be generated only if the DNA was circular. Primer specificity for the circular junction and the absence of predicted products from linear genomic DNA were confirmed in silico. Primer sequences are listed in Supplementary Table S2.

For validation, DNA was re-extracted from 1–2 mL of plasma using the same Qiagen circulating nucleic acid protocol, followed by Plasmid-Safe DNase treatment to enrich circular DNA. The enriched DNA was used as template in 50 μL PCR reactions containing 4 μL template, 0.4 μM of each primer, and a high-fidelity PCR master mix. Cycling conditions were: initial denaturation at 95 °C; 30 cycles of 98 °C for 10 s, 58 °C for 30 s, and 72 °C for 30 s; and a final extension at 72 °C for 5 min. PCR products were resolved on 2% agarose gels, and bands of the expected size (360 bp) were excised and purified using a QIAquick Gel Extraction Kit. To improve amplicon yield and sequencing reliability before Sanger confirmation, the junction-spanning PCR product was further cloned into a pUCm-T vector (Sangon Biotech, No. B522213), transformed into competent cells, screened by blue–white selection, and expanded in ampicillin-containing medium. Plasmids from positive clones were extracted, and insert identity was verified by restriction digestion. Positive clone-derived inserts were then submitted to Sangon Biotech (Shanghai) for Sanger sequencing, and the resulting sequences were aligned to the hg19 reference genome to confirm the GNA12^circle^ junction.

### qPCR validation in the large cohort

GNA12^circle^ levels in the validation cohort (109 EOPE and 109 controls) were quantified by qPCR using outward-facing primers (Supplementary Table S2). An internal spike-in control was included in each sample, by adding a known amount of circular plasmid DNA (pGEX-5X-2, absent from human plasma) at 1.2 × 10^6^ copies/mL to plasma before DNA extraction (29). DNA from validation samples was extracted using the same Qiagen kit and Plasmid-Safe DNase protocol as above. For qPCR, outward primers targeting GNA12^circle^ and a separate primer pair for the spike-in plasmid were used. Reactions contained 2 μL plasma DNA, 0.5 μM of each primer, and 5 μL of 2 × SYBR Green Master Mix (Takara). Each sample was run in triplicate for both GNA12^circle^ and the spike-in. qPCR was performed on an ABI 7500 system with the following cycling conditions: 95 °C for 5 min, followed by 40 cycles of 95 °C for 10 s and 60 °C for 30 s, plus a melt curve to verify specificity. For each sample, ΔCt values were calculated as Ct(average GNA12^circle^) – Ct(average pGEX-5X-2), and relative GNA12^circle^ abundance was derived using the 2^–ΔΔCt^ method with a control calibrator. For qPCR quality control, each sample was measured in technical triplicate for both GNA12^circle^ and the pGEX-5X-2 spike-in. Replicate-level variability was evaluated using the Ct standard deviation among triplicates. CVs were calculated from 2^-Ct^-transformed relative quantity values rather than directly from raw Ct values. As part of the experimental design, the same standard calibrator was included in triplicate in each qPCR batch to evaluate inter-assay variability. Spike-in-normalized inter-assay variability was calculated from 2^–ΔCt^ values, where ΔCt = Ct(GNA12^circle^) − Ct(spike-in). Ct-value distributions and qPCR reproducibility metrics are summarized in Supplementary Table S3.

### Predictive model construction and performance evaluation

To avoid redundancy among closely related clinical variables, body weight was not included together with BMI, and CRL was not included together with gestational age at sampling. Candidate predictors included GNA12^circle^, maternal age, maternal BMI, gestational age at sampling, NT MoM, PAPP-A MoM, and beta-hCG MoM. LASSO regression was used as a prespecified regularization and feature-selection step to determine whether any of the candidate predictors should be excluded before model training. Specifically, in each iteration of the outer five-fold cross-validation, one fold was held out as the test fold and the remaining four folds were used as the training set. All continuous predictors were standardized before LASSO regression, with scaling parameters estimated from the training set only and then applied to the held-out test fold. LASSO feature selection was performed only within the training set of that iteration, and the penalty parameter *λ* was selected by internal cross-validation within the same training set. The selected predictors were then used to train four classifiers, including logistic regression (LR), random forest (RF), extreme gradient boosting (XGB), and support vector machine (SVM). The held-out fold was not used for standardization parameter estimation, *λ* selection, feature selection, model fitting, or hyperparameter tuning, and was used only for performance evaluation. This procedure was repeated until each fold had served once as the test fold. No LASSO feature selection was performed on the full dataset before cross-validation. Although all seven prespecified candidate predictors were ultimately retained, the LASSO procedure was conducted within the cross-validation framework to avoid information leakage. Discrimination and classification performance were evaluated by the area under the receiver operating characteristic curve (AUC), accuracy, sensitivity, specificity, positive predictive value (PPV), negative predictive value (NPV), and the corresponding classification cut-off values, with 95% confidence intervals reported for all metrics.

To assess the potential impact of disease prevalence on clinical interpretation, PPV and NPV were additionally recalculated under assumed EOPE prevalences of 0.5 and 1.0% using the cross-validated mean sensitivity and specificity of the best-performing RF model. The following formulas were used: PPV = [sensitivity × prevalence] / [sensitivity × prevalence + (1 − specificity) × (1 − prevalence)] and NPV = [specificity × (1 − prevalence)] / [(1 − sensitivity) × prevalence + specificity × (1 − prevalence)]. These calculations were intended as illustrative prevalence-adjusted estimates and assumed that sensitivity and specificity remained stable across populations.

To quantify the incremental predictive contribution of GNA12^circle^, an additional clinical-only RF model was constructed by removing GNA12^circle^ and retaining the routine first-trimester variables, including maternal age, BMI, gestational age at sampling, NT MoM, PAPP-A MoM, and beta-hCG MoM. The clinical-only model was trained and evaluated using the same preprocessing procedures and five-fold cross-validation splits as the combined RF model. The AUCs of the combined and clinical-only RF models were compared using paired DeLong tests within corresponding cross-validation folds.

### Statistical analysis

Data analysis and reporting were performed in accordance with Transparent Reporting of a Multivariable Prediction Model for Individual Prognosis or Diagnosis (TRIPOD) guidelines for prediction modeling studies (Supplementary Table S4). All statistical analyses and model development were performed in R (v4.2) and Python. Continuous variables were summarized as mean ± standard deviation and median (interquartile range), as appropriate. Between-group comparisons for continuous variables were performed using Student’s t-test or the Mann–Whitney U test, as appropriate. For predictive modeling, five-fold cross-validation was used as described above, and performance metrics were summarized across folds. All tests were two-sided, and *p* < 0.05 was considered statistically significant.

## Results

### Study design and baseline characteristics of subjects

**Figure 1:**
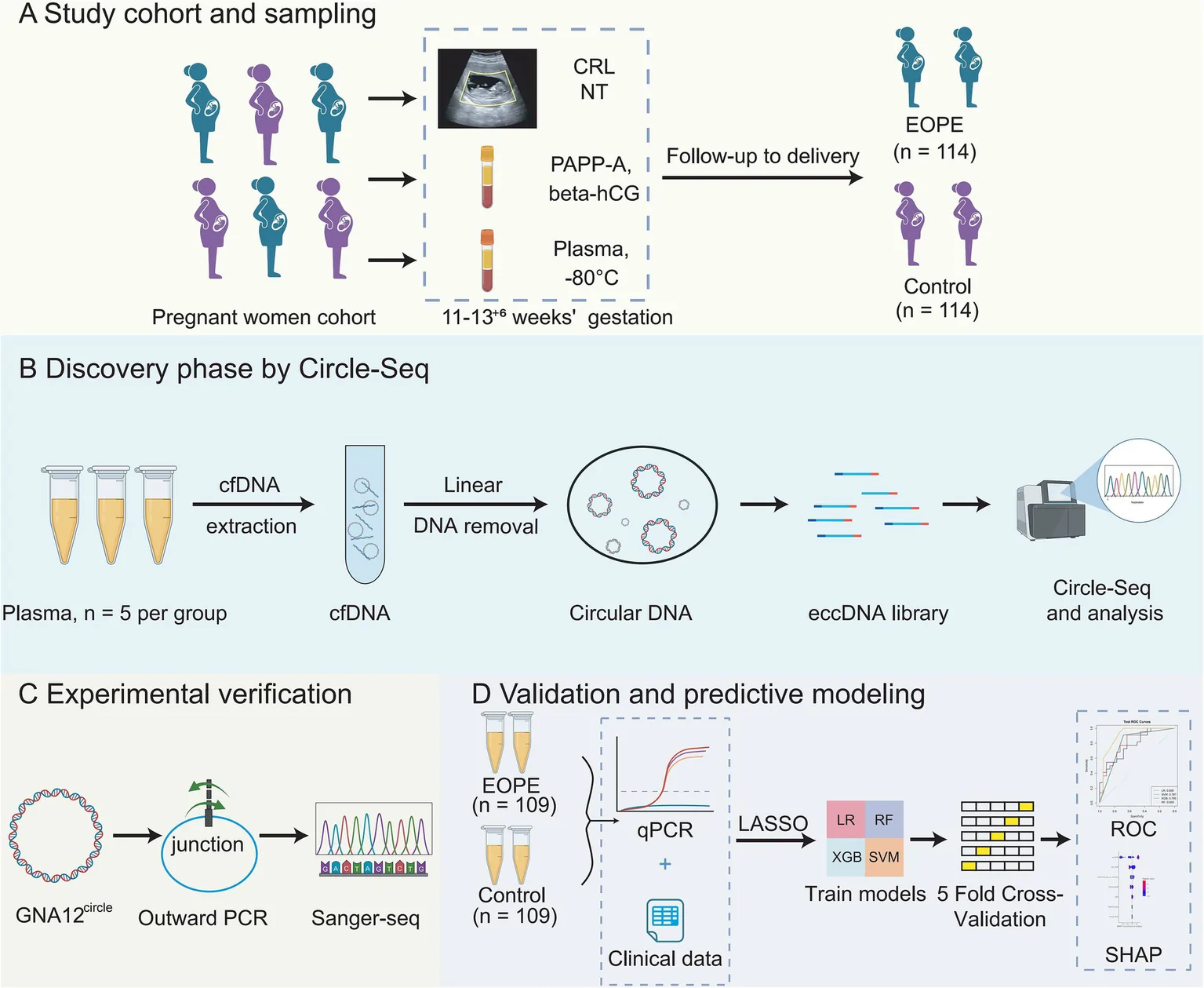
Workflow for first-trimester prediction of EOPE using plasma eccDNA. **(A)** First-trimester plasma (11–13 weeks) was collected and participants were followed to delivery; the cohort included 114 EOPE cases and 114 matched controls. **(B)** Plasma cfDNA was processed for eccDNA profiling by Circle-Seq, yielding altered eccDNAs and a candidate marker. **(C)** The candidate junction was confirmed by outward PCR and Sanger sequencing. (D) GNA12^circle^ was quantified by qPCR in the independent cohort, and prediction models were built using LASSO with LR, SVM, RF, and XGB under five-fold cross-validation; RF performed best. EOPE, early-onset preeclampsia; cfDNA, cell-free DNA; eccDNA, extrachromosomal circular DNA; LR, logistic regression; SVM, support vector machine; RF, random forest; XGB, extreme gradient boosting.

The overall workflow of sample collection, eccDNA analysis, and model development is shown in Figure 1. The baseline characteristics of the participants are summarized in Table 1. There were no significant differences in maternal age (*p* = 0.198) or height (*p* = 0.696) between groups. Women who developed EOPE had significantly higher maternal BMI and weight (*p* = 0.004 and *p* = 0.003, respectively). Additional differences were observed in crown-rump length (CRL, *p* = 0.047), and PAPP-A MoM (*p* = 0.005), while NT and beta-hCG MoM were comparable between groups.

### Plasma eccDNA profiles differ between EOPE and control pregnancies

**Figure 2:**
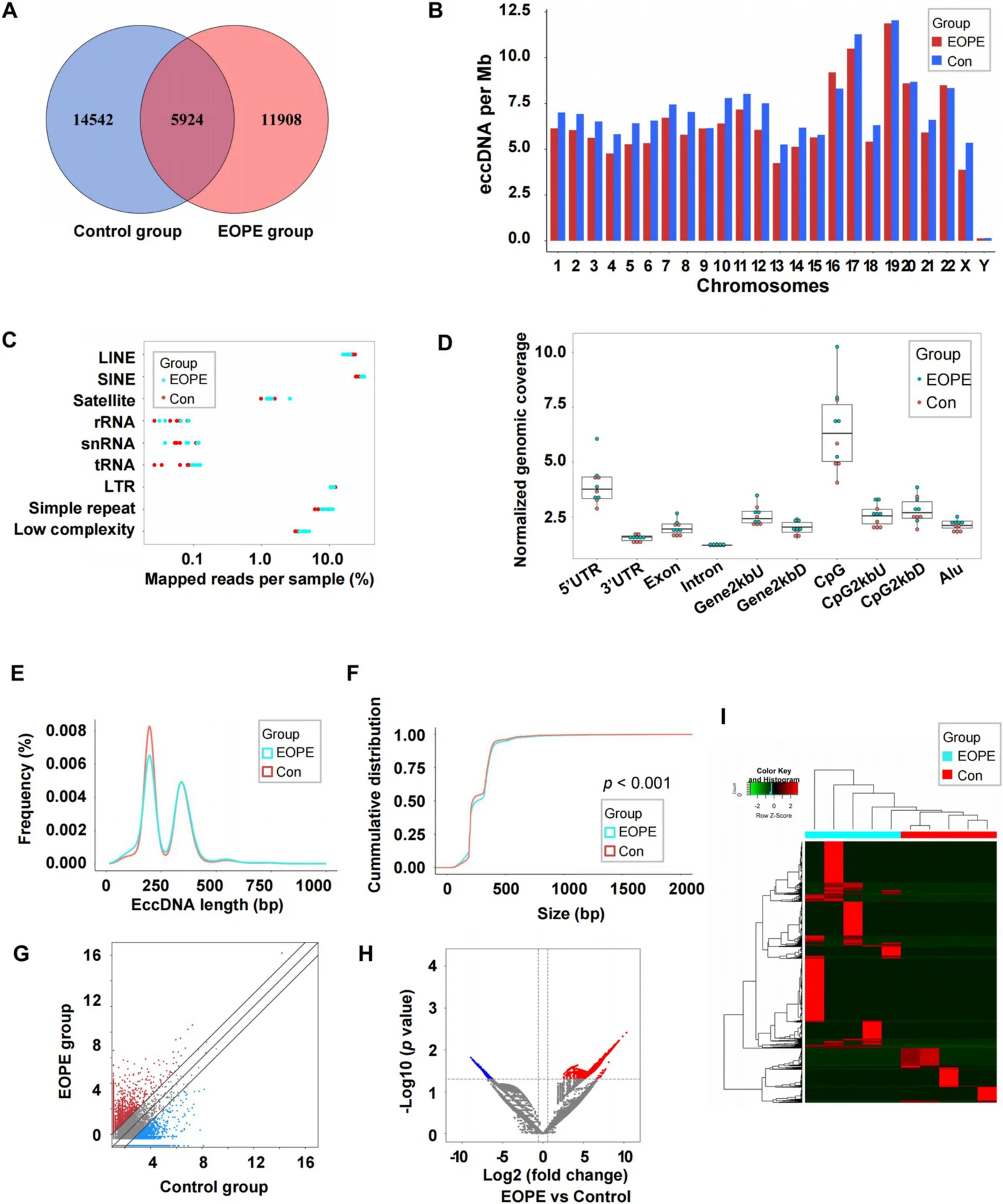
Overview of eccDNA profiles in first-trimester plasma from pregnancies with subsequent EOPE and normotensive outcomes. **(A)** Venn diagram showing the number of unique and shared eccDNA molecules between the EOPE group and the control group. **(B)** Distribution of eccDNA density (eccDNA per Mb) across chromosomes. **(C)** Composition of repetitive elements in eccDNA from the two groups, based on RepeatMasker annotation. **(D)** Normalized genomic coverage of eccDNA in various genomic regions, including 5’UTR, 3′UTR, exon, intron, gene body, CpG island, and Alu elements. **(E)** Length distribution of eccDNA, highlighting a shift in the EOPE group. **(F)** Cumulative size distribution of eccDNA in the EOPE and control groups (*p* < 0.001). **(G)** Scatter plot of eccDNA abundance between EOPE and control groups. **(H)** Volcano plot showing candidate eccDNAs with nominal differential abundance in EOPE versus control; red and blue dots represent candidate eccDNAs with nominally increased and decreased abundance, respectively, based on fold-change and nominal *p*-value thresholds (fold change ≥ 2.0 or ≤ 0.5, *p* < 0.05). **(I)** Heatmap of the 410 nominally differentially abundant candidate eccDNAs, with unsupervised hierarchical clustering of samples. EOPE, early-onset preeclampsia.

In this study, approximately 250 million reads per sample were generated using Circle-seq, with detailed quality control metrics summarized in Supplementary Table S5. Genome-wide eccDNA profiling was performed on plasma samples from women who later developed EOPE and matched controls. The sequencing data have been deposited in the GSA-Human repository^1^ under accession number HRA012499. A total of 5,924 eccDNAs were found to be shared between the two groups, with 14,542 and 11,908 unique to the control and EOPE groups, respectively (Figure 2A). EccDNA density per megabase was reduced in the EOPE group relative to controls across most chromosomes, except for autosomes 16 and 22, which exhibited higher levels (Figure 2B). EccDNA fragments detected in maternal plasma were annotated to multiple genomic features and repeat classes (Figures 2C,D). Group comparisons showed a higher representation of eccDNAs overlapping CpG islands and Alu elements in the EOPE group than in controls. Fragment length also differed between groups: eccDNAs from EOPE samples displayed a distribution shifted toward longer sizes, with a significant difference in the cumulative size distribution (*p* < 0.001, Figures 2E,F). In the exploratory discovery analysis, 410 candidate eccDNAs showed nominal differential abundance between EOPE and control samples based on fold-change and nominal *p*-value thresholds, including 264 increased and 146 decreased species in the EOPE group (Figures 2G–I). After multiple-testing correction, none of these candidate eccDNAs reached FDR < 0.05; therefore, they were interpreted as exploratory candidates rather than genome-wide significant eccDNA alterations.

### Biological function of eccDNAs

**Figure 3:**
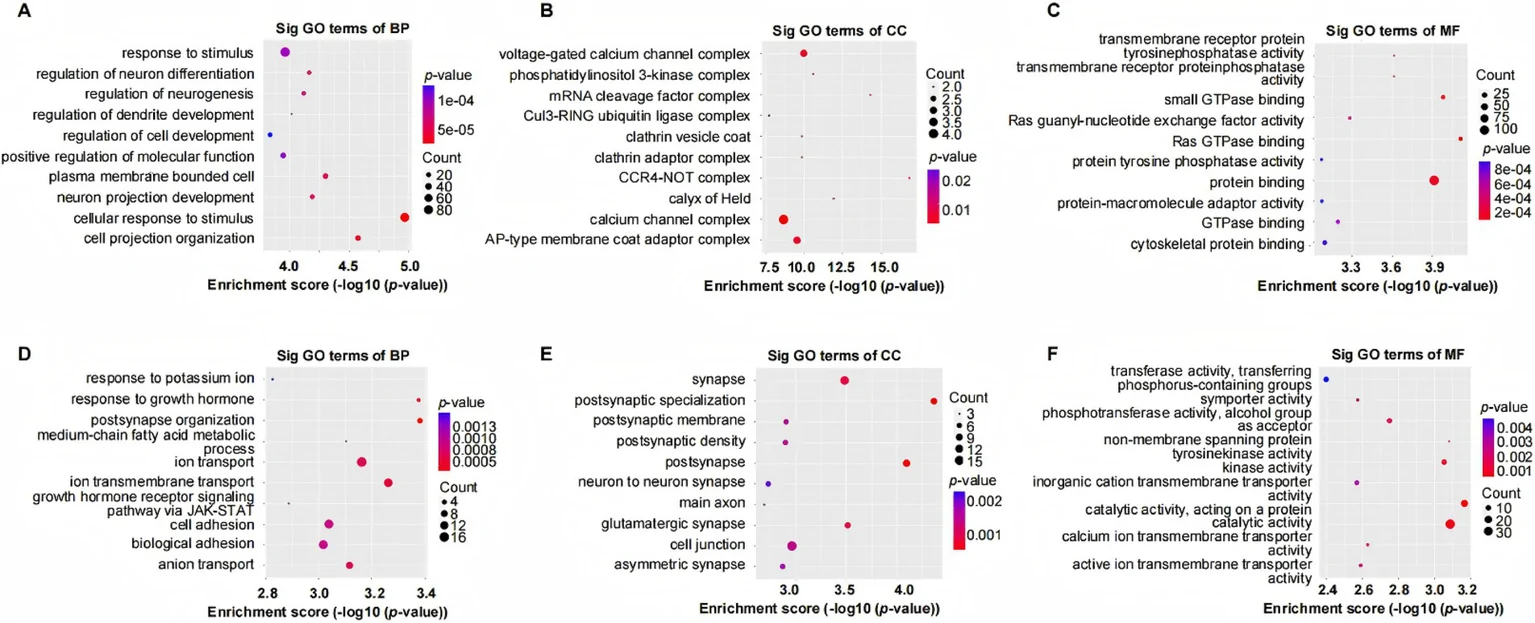
GO enrichment analysis of eccDNA-associated genes in early-onset preeclampsia (EOPE) and controls. **(A–C)** Significantly enriched Gene Ontology (GO) terms in Biological Process (BP), Cellular Component (CC), and Molecular Function (MF) categories for genes upregulated in the EOPE group. **(D–F)** Corresponding GO terms enriched in genes associated with eccDNAs showing decreased abundance in the EOPE group. The x-axis shows enrichment scores (−log₁₀ *p*-value), bubble size indicates gene count, and color reflects statistical significance (*p*-value). GO analysis was based on differentially expressed eccDNA-associated genes, using hypergeometric testing and visualized as bubble plots. GO analysis was based on genomic annotation of genes associated with candidate differentially abundant eccDNA loci and should not be interpreted as direct evidence of altered parent-gene expression.

Genes associated with eccDNAs showing increased abundance in the EOPE group were enriched in biological processes such as response to stimulus, neuron differentiation, and cell projection organization. These eccDNA-associated genes were mainly localized to calcium channel complexes, clathrin-coated vesicles, and AP-type membrane coat adaptor complexes, and were involved in molecular functions including transmembrane receptor activity, GTPase binding, and cytoskeletal protein binding (Figures 3A–C). In contrast, genes associated with eccDNAs showing decreased abundance in the EOPE group were enriched in processes such as potassium ion response, JAK–STAT signaling, and cell adhesion. They were localized to synaptic structures including the postsynaptic membrane, asymmetric synapse, and main axon, and were associated with molecular functions such as kinase activity, ion transporter activity, and protein catalytic activity (Figures 3D–F). Selected signaling pathways with potential relevance to EOPE, annotated from genes associated with candidate differentially abundant eccDNA loci, are summarized in Table 2.

**Table 2:** The significantly enriched preeclampsia-related signaling pathways for the altered eccDNAs.

Term	Regulation	Count	*p* value	Enrichment score
Leukocyte transendothelial migration	Up	5	0.002	2.651
Insulin signaling pathway	Up	5	0.005	2.344
Rap1 signaling pathway	Up	6	0.006	2.225
Type II diabetes mellitus	Up	3	0.006	2.207
VEGF signaling pathway	Up	3	0.012	1.920
MAPK signaling pathway	Up	6	0.014	1.841
Natural killer cell mediated cytotoxicity	Up	4	0.021	1.673
Insulin secretion	Up	3	0.029	1.539
AMPK signaling pathway	Down	3	0.019	1.731
Adipocytokine signaling pathway	Down	2	0.040	1.393
Chemokine signaling pathway	Down	3	0.053	1.276
Insulin resistance	Down	2	0.088	1.054
MAPK signaling pathway	Down	3	0.110	0.960

Term represents the pathway name. Count refers to the number of selected eccDNAs mapped to each corresponding pathway. eccDNA, extrachromosomal circular DNA; VEGF, vascular endothelial growth factor; MAPK, mitogen-activated protein kinase; AMPK, AMP-activated protein kinase.

### GNA12^circle^ is significantly elevated in women who developed EOPE

**Figure 4:**
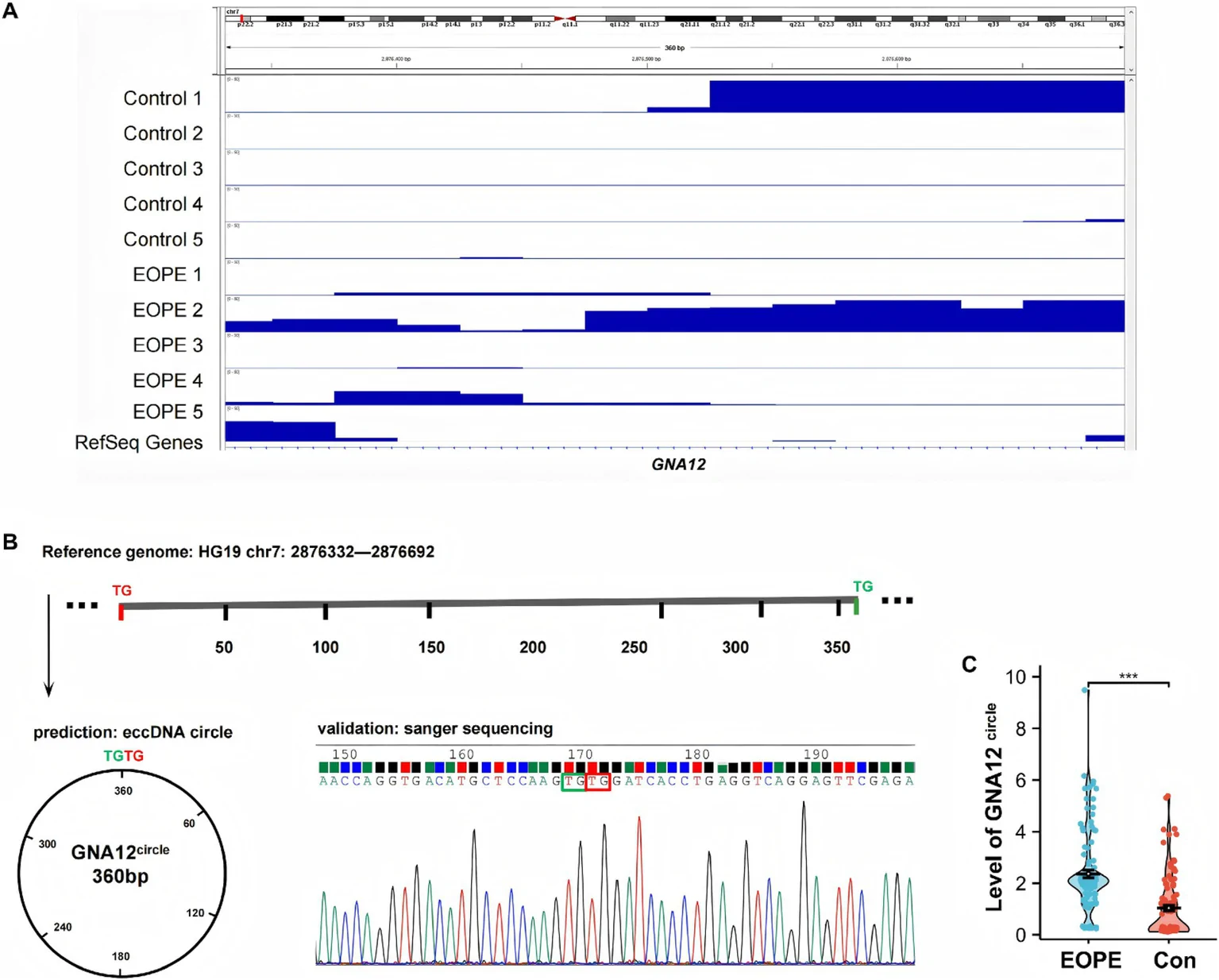
Identification and validation of GNA12^circle^ as an EOPE-associated eccDNA. **(A)** Visualization of eccDNA reads aligned to the *GNA12* gene locus on chromosome 7 using genome browser tracks, showing stronger GNA12^circle^ signal in EOPE samples compared to controls. **(B)** Schematic diagram of the predicted circular DNA formed by a 360 bp genomic segment within the *GNA12* locus. Breakpoint junction was confirmed by Sanger sequencing, with representative chromatogram and aligned sequence. **(C)** Violin plot showing increased GNA12^circle^ levels in early pregnancy plasma from pregnancies that subsequently developed EOPE, as measured by quantitative PCR (***p < 0.001). EOPE, early-onset preeclampsia; eccDNA, extrachromosomal circular DNA.

Among the nominally differentially abundant eccDNAs, a circular DNA derived from the *GNA12* gene locus on chromosome 7 (chr7:2876332–2,876,692; GNA12^circle^) was nominated as an exploratory candidate for orthogonal validation. GNA12^circle^ was not selected as an FDR-significant genome-wide hit, but rather as a prioritized candidate from the *p*-value-screened exploratory pool. Candidate nomination considered nominal enrichment in EOPE samples, detection pattern across discovery samples, fragment length suitable for outward PCR and junction-specific qPCR, feasibility of designing junction-spanning primers, and biological plausibility of the corresponding genomic locus. GNA12^circle^ was retained for large-cohort validation because it showed nominal enrichment in EOPE samples (logFC = 6.81, *p* = 0.025; FDR = 0.146), had a 360 bp fragment length suitable for outward PCR and junction-specific qPCR, and yielded a reproducible junction-spanning PCR product that was confirmed by Sanger sequencing. As shown in Figure 4A, Integrative Genomics Viewer (IGV) tracks revealed a higher abundance of GNA12^circle^ in the EOPE group compared to the control group. Outward PCR using primers specific to the junction region was performed, and the circular structure of GNA12^circle^ was confirmed by Sanger sequencing (Figure 4B). Consistent with the sequencing results, qPCR showed significantly higher GNA12^circle^ abundance in the EOPE group than in controls (n = 109 per group, *p* < 0.001; Figure 4C). The junction-specific qPCR assay showed acceptable analytical reproducibility. All technical triplicates from the 218 validation samples had Ct SD ≤ 0.5 cycles. The median intra-assay CVs were 7.74% for GNA12^circle^ and 5.27% for the pGEX-5X-2 spike-in, and the spike-in-normalized inter-assay CV based on the standard calibrator was 6.19% (Supplementary Table S3).

### Machine learning models showed good discrimination for EOPE prediction

**Figure 5:**
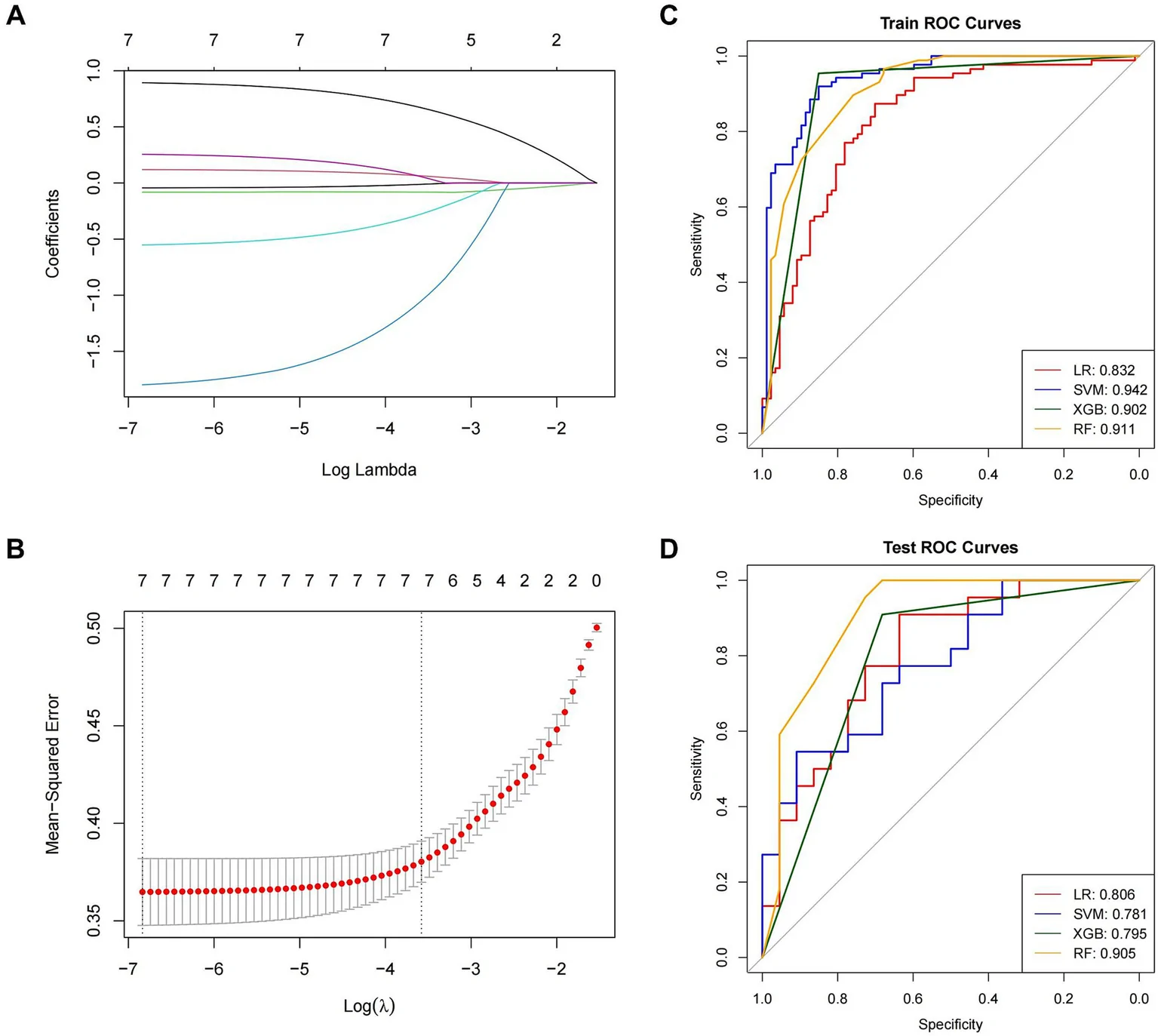
Feature selection and model performance evaluation for EOPE prediction. **(A)** LASSO coefficient profiles of candidate variables plotted against the L1 regularization path. **(B)** Five-fold cross-validation curve used to select the optimal *λ* value in LASSO regression. The dotted line indicates the minimum mean squared error and corresponding *λ*. **(C)** ROC curves of four machine learning models—LR, SVM, XGB, and RF—in the training folds of a representative cross-validation iteration. **(D)** ROC curves of the same models in the corresponding representative held-out test fold. The AUC values shown in the legend are fold-specific values for this representative fold and should not be interpreted as the overall cross-validated mean AUC. EOPE, early-onset preeclampsia; ROC, receiver operating characteristic; LR, logistic regression; SVM, support vector machine; XGB, extreme gradient boosting; RF, random forest; AUC, area under the curve.

Feature selection was performed using LASSO regression within the training folds of the cross-validation procedure. LASSO did not exclude any of the seven prespecified candidate predictors; therefore, GNA12^circle^, maternal age, BMI, gestational age at sampling, NT MoM, PAPP-A MoM, and beta-hCG MoM were retained for downstream modeling (Figures 5A,B). The retained predictors and their penalized coefficients are presented in Table 3. No feature selection was performed on the full dataset before cross-validation. Using these selected variables, four classifiers were then developed for EOPE prediction, including LR, SVM, XGB, and RF. Across the evaluated classifiers, RF showed the best overall cross-validated discrimination, with a mean held-out test-fold AUC of 0.843 across the five cross-validation folds. The representative ROC curves are shown in Figures 5C,D, where the RF model achieved an AUC of 0.905 in the displayed representative held-out test fold. Fold-specific cross-validated performance metrics are summarized in Supplementary Figure S1 and Table 4.

**Table 3:** Predictors retained by LASSO regression and their penalized coefficients.

Variable	LASSO coefficient
Maternal age	−0.0191
BMI	0.0759
Gestational age at sampling	−0.0805
NT MoM	−1.2161
PAPPA MoM	−0.3363
beta-hCG MoM	0.1019
GNA12^circle^	0.7173

**Table 4:** Cross-validated performance of prediction models for early-onset preeclampsia.

Fold	Set	Model	AUC(95%CI)	Accuracy(95%CI)	Sensitivity(95%CI)	Specificity(95%CI)	PPV(95%CI)	NPV(95%CI)	Cut-off
1	Train	LR	0.835 (0.774–0.895)	0.777 (0.708–0.836)	0.655 (0.555–0.755)	0.898 (0.834–0.961)	0.864 (0.781–0.946)	0.725 (0.641–0.809)	0.375
1	Test	LR	0.781 (0.638–0.924)	0.767 (0.614–0.882)	0.864 (0.720–1.000)	0.667 (0.465–0.868)	0.731 (0.560–0.901)	0.824 (0.642–1.000)	0.375
2	Train	LR	0.845 (0.785–0.905)	0.799 (0.732–0.856)	0.770 (0.682–0.859)	0.828 (0.748–0.907)	0.817 (0.733–0.901)	0.783 (0.698–0.867)	0.442
2	Test	LR	0.781 (0.642–0.920)	0.727 (0.572–0.850)	0.545 (0.337–0.754)	0.909 (0.789–1.000)	0.857 (0.674–1.000)	0.667 (0.498–0.835)	0.442
3	Train	LR	0.842 (0.784–0.901)	0.794 (0.727–0.852)	0.773 (0.685–0.860)	0.816 (0.735–0.897)	0.810 (0.726–0.893)	0.780 (0.695–0.865)	0.476
3	Test	LR	0.788 (0.633–0.943)	0.837 (0.693–0.932)	0.667 (0.465–0.868)	1.000 (1.000–1.000)	1.000 (1.000–1.000)	0.759 (0.603–0.914)	0.476
4	Train	LR	0.832 (0.771–0.893)	0.787 (0.719–0.846)	0.701 (0.605–0.797)	0.874 (0.804–0.943)	0.847 (0.764–0.930)	0.745 (0.661–0.830)	0.413
4	Test	LR	0.835 (0.705–0.965)	0.818 (0.673–0.918)	0.909 (0.789–1.000)	0.727 (0.541–0.913)	0.769 (0.607–0.931)	0.889 (0.744–1.000)	0.413
5	Train	LR	0.832 (0.770–0.894)	0.787 (0.719–0.846)	0.701 (0.605–0.797)	0.874 (0.804–0.943)	0.847 (0.764–0.930)	0.745 (0.661–0.830)	0.379
5	Test	LR	0.806 (0.676–0.936)	0.773 (0.622–0.885)	0.636 (0.435–0.837)	0.909 (0.789–1.000)	0.875 (0.713–1.000)	0.714 (0.547–0.882)	0.379
1	Train	RF	0.881 (0.832–0.930)	0.800 (0.733–0.857)	0.736 (0.643–0.828)	0.864 (0.792–0.935)	0.842 (0.760–0.924)	0.768 (0.684–0.851)	0.525
1	Test	RF	0.850 (0.734–0.965)	0.767 (0.614–0.882)	0.818 (0.657–0.979)	0.714 (0.521–0.908)	0.750 (0.577–0.923)	0.789 (0.606–0.973)	0.525
2	Train	RF	0.910 (0.869–0.952)	0.833 (0.769–0.885)	0.793 (0.708–0.878)	0.874 (0.804–0.943)	0.863 (0.787–0.938)	0.809 (0.729–0.888)	0.475
2	Test	RF	0.798 (0.663–0.932)	0.682 (0.524–0.814)	0.591 (0.385–0.796)	0.773 (0.598–0.948)	0.722 (0.515–0.929)	0.654 (0.471–0.837)	0.475
3	Train	RF	0.908 (0.865–0.952)	0.851 (0.790–0.901)	0.750 (0.660–0.840)	0.954 (0.910–0.998)	0.943 (0.888–0.997)	0.790 (0.713–0.868)	0.425
3	Test	RF	0.775 (0.625–0.925)	0.744 (0.588–0.865)	0.619 (0.411–0.827)	0.864 (0.720–1.000)	0.812 (0.621–1.000)	0.704 (0.531–0.876)	0.425
4	Train	RF	0.889 (0.842–0.937)	0.816 (0.750–0.871)	0.724 (0.630–0.818)	0.908 (0.847–0.969)	0.887 (0.814–0.961)	0.767 (0.685–0.849)	0.625
4	Test	RF	0.885 (0.788–0.983)	0.750 (0.597–0.868)	0.636 (0.435–0.837)	0.864 (0.720–1.000)	0.824 (0.642–1.000)	0.704 (0.531–0.876)	0.625
5	Train	RF	0.911 (0.868–0.954)	0.828 (0.763–0.881)	0.759 (0.669–0.849)	0.897 (0.833–0.961)	0.880 (0.806–0.954)	0.788 (0.707–0.868)	0.675
5	Test	RF	0.905 (0.810–1.000)	0.795 (0.647–0.902)	0.864 (0.720–1.000)	0.727 (0.541–0.913)	0.760 (0.593–0.927)	0.842 (0.678–1.000)	0.675
1	Train	XGB	0.903 (0.859–0.946)	0.903 (0.849–0.942)	0.851 (0.776–0.925)	0.955 (0.911–0.998)	0.949 (0.900–0.998)	0.866 (0.798–0.934)	0.5
1	Test	XGB	0.766 (0.637–0.895)	0.767 (0.614–0.882)	0.818 (0.657–0.979)	0.714 (0.521–0.908)	0.750 (0.577–0.923)	0.789 (0.606–0.973)	0.5
2	Train	XGB	0.908 (0.865–0.951)	0.908 (0.855–0.947)	0.862 (0.790–0.935)	0.954 (0.910–0.998)	0.949 (0.901–0.998)	0.874 (0.807–0.940)	0.5
2	Test	XGB	0.795 (0.678–0.913)	0.795 (0.647–0.902)	0.682 (0.487–0.876)	0.909 (0.789–1.000)	0.882 (0.729–1.000)	0.741 (0.575–0.906)	0.5
3	Train	XGB	0.909 (0.867–0.951)	0.909 (0.856–0.947)	0.852 (0.778–0.926)	0.966 (0.927–1.000)	0.962 (0.919–1.000)	0.866 (0.798–0.934)	0.5
3	Test	XGB	0.765 (0.638–0.892)	0.767 (0.614–0.882)	0.667 (0.465–0.868)	0.864 (0.720–1.000)	0.824 (0.642–1.000)	0.731 (0.560–0.901)	0.5
4	Train	XGB	0.914 (0.873–0.955)	0.914 (0.862–0.951)	0.862 (0.790–0.935)	0.966 (0.927–1.000)	0.962 (0.919–1.000)	0.875 (0.809–0.941)	0.5
4	Test	XGB	0.773 (0.649–0.896)	0.773 (0.622–0.885)	0.682 (0.487–0.876)	0.864 (0.720–1.000)	0.833 (0.661–1.000)	0.731 (0.560–0.901)	0.5
5	Train	XGB	0.902 (0.859–0.946)	0.902 (0.848–0.942)	0.851 (0.776–0.925)	0.954 (0.910–0.998)	0.949 (0.900–0.998)	0.865 (0.796–0.933)	0.5
5	Test	XGB	0.795 (0.678–0.913)	0.795 (0.647–0.902)	0.682 (0.487–0.876)	0.909 (0.789–1.000)	0.882 (0.729–1.000)	0.741 (0.575–0.906)	0.5
1	Train	SVM	0.932 (0.897–0.968)	0.874 (0.816–0.920)	0.862 (0.790–0.935)	0.886 (0.820–0.953)	0.882 (0.814–0.951)	0.867 (0.796–0.937)	0.499
1	Test	SVM	0.872 (0.763–0.981)	0.791 (0.640–0.900)	0.909 (0.789–1.000)	0.667 (0.465–0.868)	0.741 (0.575–0.906)	0.875 (0.713–1.000)	0.499
2	Train	SVM	0.946 (0.914–0.979)	0.885 (0.828–0.928)	0.862 (0.790–0.935)	0.908 (0.847–0.969)	0.904 (0.840–0.967)	0.868 (0.799–0.938)	0.539
2	Test	SVM	0.795 (0.664–0.927)	0.727 (0.572–0.850)	0.591 (0.385–0.796)	0.864 (0.720–1.000)	0.812 (0.621–1.000)	0.679 (0.506–0.852)	0.539
3	Train	SVM	0.953 (0.926–0.980)	0.880 (0.822–0.924)	0.807 (0.724–0.889)	0.954 (0.910–0.998)	0.947 (0.896–0.998)	0.830 (0.756–0.904)	0.377
3	Test	SVM	0.771 (0.614–0.927)	0.767 (0.614–0.882)	0.667 (0.465–0.868)	0.864 (0.720–1.000)	0.824 (0.642–1.000)	0.731 (0.560–0.901)	0.377
4	Train	SVM	0.942 (0.909–0.975)	0.874 (0.815–0.919)	0.793 (0.708–0.878)	0.954 (0.910–0.998)	0.945 (0.893–0.997)	0.822 (0.747–0.896)	0.469
4	Test	SVM	0.837 (0.715–0.958)	0.750 (0.597–0.868)	0.682 (0.487–0.876)	0.818 (0.657–0.979)	0.789 (0.606–0.973)	0.720 (0.544–0.896)	0.469
5	Train	SVM	0.942 (0.909–0.976)	0.885 (0.828–0.928)	0.851 (0.776–0.925)	0.920 (0.862–0.977)	0.914 (0.852–0.975)	0.860 (0.790–0.931)	0.481
5	Test	SVM	0.781 (0.647–0.915)	0.682 (0.524–0.814)	0.636 (0.435–0.837)	0.727 (0.541–0.913)	0.700 (0.499–0.901)	0.667 (0.478–0.855)	0.481

To further assess the incremental predictive value of GNA12^circle^, we compared the combined RF model with a clinical-only RF model that excluded GNA12^circle^. Across the five held-out test folds, the clinical-only RF model showed lower discrimination than the combined RF model, with a mean test-fold AUC of 0.639 ± 0.054 compared with 0.843 ± 0.055 for the combined model. The mean increase in AUC after adding GNA12^circle^ was 0.204. Paired DeLong tests comparing the two models within corresponding held-out test folds showed statistically significant differences across folds. The fold-specific comparison is provided in Supplementary Table S6.

### Model calibration and clinical utility

**Figure 6:**
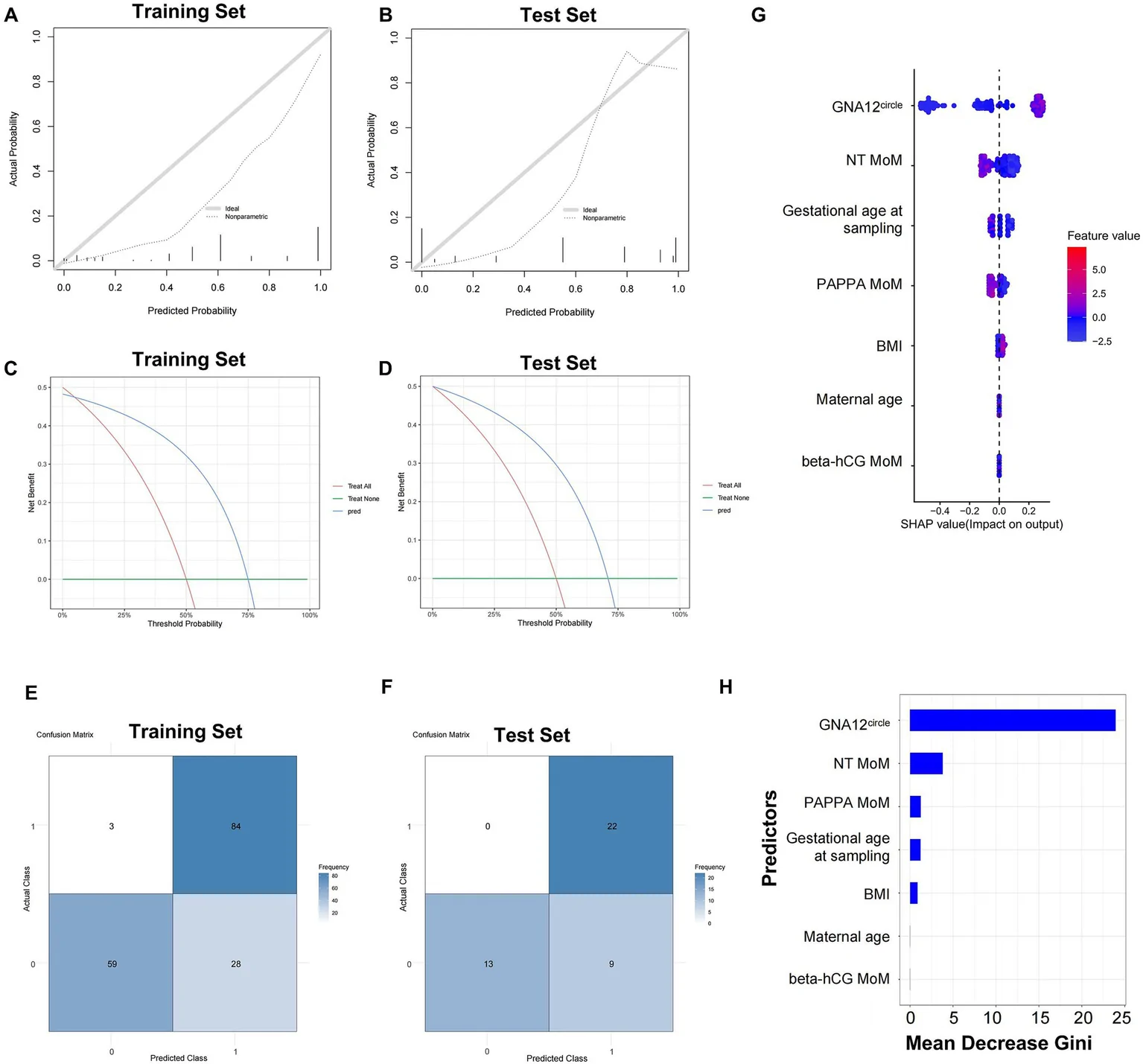
Model calibration, clinical utility, and feature importance in predicting EOPE. **(A,B)** Calibration plots in the training set **(A)** and test set **(B)**, comparing predicted probabilities with actual outcomes. **(C,D)** Decision curve analysis (DCA) showing net benefit across threshold probabilities in the training **(C)** and test sets **(D)**. **(E,F)** Confusion matrices illustrating model classification performance in training **(E)** and test sets **(F)**. **(G)** SHAP summary plot showing the contribution of each predictor to the RF model output. The color scale represents feature values. **(H)** Global variable importance of the RF model ranked by mean decrease in Gini impurity. BMI, body mass index; NT, nuchal translucency; MoM, multiples of the median.

The calibration plot of the held-out test fold suggested reasonable overall agreement between predicted and observed probabilities, but noticeable deviation from the ideal diagonal was observed at higher predicted probabilities (approximately >0.6), indicating less stable calibration in the high-risk range (Figures 6A,B). Decision-curve analysis indicated that the model provided a higher net benefit than “treat all” or “treat none” across clinically relevant threshold probabilities (Figures 6C,D). Confusion matrices indicated high sensitivity but modest specificity, particularly in the test set (Figures 6E,F).

Because the validation cohort used a 1:1 case–control design, we further estimated prevalence-adjusted predictive values for the best-performing RF model. Using the cross-validated mean sensitivity of 0.706 and specificity of 0.788, the adjusted PPV was estimated to be 3.2% when EOPE prevalence was assumed to be 1.0%, with an adjusted NPV of 99.6%. When EOPE prevalence was assumed to be 0.5%, the adjusted PPV decreased to 1.6%, with an adjusted NPV of 99.8% (Supplementary Table S7). These estimates indicate that the model’s apparent predictive value in the case–control setting should not be directly extrapolated to population-based screening.

SHAP-based analysis provided a complementary assessment of feature contribution to the final RF model output (Figure 6G). In the SHAP summary plot, GNA12^circle^ appeared as the top-ranked predictor and showed the broadest contribution pattern among the included variables, indicating a prominent contribution to individual-level RF model predictions. In the RF model, GNA12^circle^ showed the largest mean decrease in Gini impurity, indicating its strong contribution to node splitting and impurity reduction (Figure 6H). These findings suggest that GNA12^circle^ provided important predictive information while also highlighting the complementary contribution of routine clinical variables.

## Discussion

We first examined first-trimester plasma eccDNA profiles in pregnancies that later developed EOPE and found that they differed from those of normotensive controls. GNA12^circle^ was then selected from the profiling results for further evaluation and validated as a candidate circulating marker. Plasma GNA12^circle^ levels were higher at 11–13 weeks in women who subsequently developed EOPE. In our cohort, comparison with a clinical-only RF model suggested that adding GNA12^circle^ improved discrimination beyond routinely available first-trimester variables. Among the models we tested, random forest showed the best discrimination. Taken together, these findings suggest that first-trimester plasma eccDNA may provide complementary information for identifying pregnancies at increased risk of EOPE.

Our results support ongoing efforts to improve first-trimester prediction of EOPE with models that are feasible in routine care. Established approaches, most notably the FMF competing-risk framework, integrate maternal characteristics with mean arterial pressure, uterine artery Doppler indices, and placental biomarkers to identify women at high risk (30, 31). Prospective studies in nulliparous populations suggest that these strategies can detect a substantial proportion of EOPE or preterm PE, although false-positive rates remain non-trivial (11). In parallel, recent studies have explored additional molecular markers for early risk stratification, including angiogenic biomarkers and multi-omics signals such as cell-free RNA and cell-free DNA methylation profiles (32–35). These approaches may help identify pregnancies at risk of EOPE, but many still depend on specialized assays or technical platforms. Our approach was kept relatively simple. It used a limited number of routinely available first-trimester variables together with one targeted eccDNA marker. Most of the required information is already collected during standard first-trimester care. The only added measurement is a junction-specific qPCR assay for the eccDNA target. Compared with protocols that rely heavily on Doppler assessment, this workflow may be easier to apply across different settings. Some recent studies found that adding maternal hemodynamic measures to first-trimester screening brought only limited improvement over existing approaches (36). In our model interpretation analyses, the eccDNA signal still contributed additional information to EOPE risk stratification.

Circulating nucleic acids are being used more often as liquid-biopsy analytes to study placental biology, partly following the clinical success of cell-free DNA in prenatal screening (13). Within this broader group of analytes, eccDNA represents a distinct form of circulating cell-free DNA. Its covalently closed structure may confer some resistance to exonuclease degradation. Sequencing-based workflows can profile eccDNA without predefined targets, which is useful for biomarker discovery (16, 37). In PE, evidence for a circulating eccDNA signal is beginning to emerge. Plasma eccDNA profiling in established PE has shown disease-associated shifts in eccDNA landscapes, along with a higher eccDNA burden than that seen in normotensive pregnancies. This suggests that eccDNA alterations can be detected in maternal circulation (38). In our cohort, plasma eccDNA levels were already lower at 11–13 weeks in pregnancies that later developed EOPE. Our study did not stop at descriptive profiling. We also examined whether an eccDNA signal could contribute to early risk assessment in a clinically relevant setting. We found a candidate circulating eccDNA marker at 11–13 weeks, then evaluated it in an independent cohort. It was also assessed together with standard first-trimester variables in multivariable models for EOPE. This discovery to validation approach lines up with reports from other placenta mediated complications where eccDNA profiles showed biomarker potential in early pregnancy and in placental dysfunction settings (21, 22). These findings support eccDNA as a potentially informative molecular layer for first-trimester EOPE risk stratification. Further validation will still be needed before clinical use.

A key question is why GNA12^circle^ showed predictive relevance for EOPE in our models. *GNA12* encodes Gα12 subunit of heterotrimeric G proteins, which participates in signaling pathways related to cytoskeletal remodeling, cell motility, and cellular stress responses, processes that are relevant to trophoblast invasion and maternal endothelial dysfunction (1, 39). Although *GNA12* is not a canonical preeclampsia biomarker, previous epigenetic and transcriptional studies suggest that this locus may be dysregulated in PE. Reduced methylation at the *GNA12* promoter and increased *GNA12* mRNA expression have been reported in placental tissue from PE pregnancies, and similar methylation signals have also been detected in maternal blood DNA (40). In addition, translational placental profiling identified *GNA12* overexpression in one molecular subset of PE, suggesting that *GNA12*-related signaling may represent a pathophysiologic axis that is not fully captured by traditional angiogenic markers (41). Based on these observations, elevated first-trimester GNA12^circle^ may be consistent with early placental stress, altered trophoblast turnover, or release of eccDNA from epigenetically active genomic loci, but this interpretation remains speculative. However, whether circulating GNA12^circle^ is merely a marker of disease-related cellular stress or has a functional role in EOPE pathogenesis remains unknown. Recent work suggests that eccDNA can trigger innate immune sensing pathways such as STING, offering a possible but unproven connection to inflammatory signaling in PE (25). Future studies incorporating tissue-of-origin analysis and functional experiments will be required to clarify the biological source of GNA12^circle^, the triggers of eccDNA generation, and its potential effects in placental and endothelial models.

A strength of this study is its two-stage design. We first used unbiased eccDNA sequencing for marker discovery and then evaluated the selected signal in an independent validation cohort. This design helps reduce false-positive findings and provides more preliminary support for the association of GNA12^circle^ with EOPE. We also did not assess the eccDNA marker as a standalone test. Instead, it was incorporated into multivariable models together with routinely available first-trimester predictors. This allowed us to examine its added value beyond conventional parameters such as maternal BMI and PAPP-A, whose predictive performance alone is often limited (42, 43). In our analyses, model interpretation supported an independent contribution of the eccDNA signal. GNA12^circle^ ranked among the most informative predictors, suggesting that it captures risk information not fully reflected by conventional factors. Earlier identification may support timely preventive strategies, such as low-dose aspirin before 16 weeks of gestation, and closer surveillance when appropriate (5, 7).

Several limitations merit consideration. First, this was a single-center study, and the sequencing-based discovery phase included only 5 EOPE cases and 5 controls. In addition, eccDNA discovery differs from conventional gene-expression analysis because eccDNA identification and quantification depend mainly on junction-spanning reads, resulting in sparse count matrices. Although FDR values were calculated and reported for transparency, no candidate eccDNA remained significant at FDR < 0.05. Therefore, the 410 candidate eccDNAs should be interpreted as nominally differentially abundant, hypothesis-generating signals rather than definitive genome-wide significant loci. Larger multicenter discovery cohorts and statistical methods optimized for sparse eccDNA junction-read data are needed to establish robust eccDNA signatures for EOPE.

Second, the 1:1 case–control design may overestimate apparent predictive value and clinical screening performance compared with an unselected screening population in which EOPE prevalence is low. In our prevalence-adjusted analysis based on the sensitivity and specificity of the corrected RF model, the estimated PPV decreased substantially under assumed EOPE prevalences of 1.0 and 0.5%, although the corresponding NPV remained high. Therefore, the present model should be interpreted as a candidate risk-stratification tool rather than a clinically ready population screening test. Future prospective cohort studies should evaluate discrimination, calibration, PPV, NPV, and decision-curve utility under real-world prevalence, with appropriate recalibration and threshold optimization.

Third, this study did not establish causality or conclusively determine whether circulating GNA12^circle^ is maternal or placental in origin. Follow-up studies incorporating tissue-of-origin signatures and mechanistic experiments in trophoblast and endothelial systems are needed to clarify the biological source and functional relevance of this signal. In addition, only one prioritized candidate eccDNA was advanced to large-cohort qPCR validation in this proof-of-concept study. Future studies should validate multiple prioritized candidates to determine whether additional eccDNA markers have independent or complementary predictive value. The GO enrichment analysis should also be interpreted cautiously, because eccDNA circularization does not necessarily indicate altered expression or functional activation of the parent gene.

Finally, additional assay-standardization experiments remain necessary before clinical implementation. Although we added Ct-value distributions and intra−/inter-assay reproducibility metrics based on technical triplicates and the common standard calibrator, further analytical validation remains necessary before clinical implementation, including plasmid-based serial dilution standard curves, amplification efficiency assessment, LOD, LOQ, formal repeated-run and inter-laboratory reproducibility testing, convergent-primer controls, genomic DNA negative controls, and preferably ddPCR-based validation. Junction-specific qPCR is feasible, but reproducibility, thresholds, batch effects, and cost must be rigorously evaluated. Longitudinal sampling may further clarify temporal trajectories and determine whether eccDNA dynamics track disease severity or refine the optimal prediction window.

## Conclusion

This study provides preliminary evidence that first-trimester plasma eccDNA may provide complementary information for early risk stratification of EOPE. Using a discovery-to-validation strategy, we identified and independently confirmed a candidate circulating eccDNA marker that was elevated at 11–13 weeks in pregnancies that later developed EOPE. Integrating this targeted eccDNA measurement with routinely collected first-trimester variables yielded prediction models with encouraging performance. These results support eccDNA as a candidate molecular complement to conventional risk factors. Further validation in larger, multicenter prospective cohorts and standardization of junction-specific qPCR assays will be required before clinical implementation. Overall, our findings support further development of eccDNA-informed, workflow-compatible approaches to refine early-pregnancy risk assessment.

## Data Availability

The datasets presented in this study can be found in online repositories. The names of the repository/repositories and accession number(s) can be found in the article/Supplementary material.

## Footnotes

1
https://ngdc.cncb.ac.cn/gsa-human

## Supplementary material

Supplementary Figure S1 Performance comparison of machine learning models across cross-validation folds. **(A)** AUC with 95% confidence intervals for each model (LR, RF, SVM, XGB) across five cross-validation folds, shown separately for test and training sets. **(B)** Bar plots showing AUC values for each fold per model, with one representative fold highlighted in black and its corresponding AUC and 95% CI displayed. LR, logistic regression; RF, random forest; SVM, support vector machine; XGB, extreme gradient boosting; AUC, area under the ROC curve; CI, confidence interval.

## Glossary

ACOG: American College of Obstetricians and Gynecologists
AMPK: AMP-activated protein kinase
ATP: Adenosine triphosphate
AUC: Area under the receiver operating characteristic curve
BEDTools: A toolkit for genome feature analysis
BMI: Body mass index
BP: Biological Process (Gene Ontology category)
BWA-MEM: Burrows–Wheeler Aligner maximal exact matches algorithm
CC: Cellular Component (Gene Ontology category)
cfDNA: Cell-free DNA
CI: Confidence interval
Circle-Seq: Sequencing-based workflow for profiling extrachromosomal circular DNA
CRL: Crown–rump length
Ct: Cycle threshold
DCA: Decision curve analysis
DNase: Deoxyribonuclease
eccDNA: Extrachromosomal circular DNA
EDTA: Ethylenediaminetetraacetic acid
EOPE: Early-onset preeclampsia
FMF: Fetal Medicine Foundation
GO: Gene Ontology
GRCh37: Genome Reference Consortium Human Build 37
GSA: Genome Sequence Archive
hg19: Human Genome version 19 (reference build)
IGV: Integrative Genomics Viewer
IRB: Institutional Review Board
JAK–STAT: Janus kinase–signal transducer and activator of transcription
LASSO: Least absolute shrinkage and selection operator
LR: Logistic regression
MAPK: Mitogen-activated protein kinase
MF: Molecular Function (Gene Ontology category)
MoM: Multiples of the median
NPV: Negative predictive value
NT: Nuchal translucency
PAPP-A: pregnancy-associated plasma protein-A
PCR: Polymerase chain reaction
PE: Preeclampsia
PlGF: Placental growth factor
PPV: Positive predictive value
Q30: Phred quality score of 30
qPCR: Quantitative polymerase chain reaction
RF: Random forest
ROC: Receiver operating characteristic
sFlt-1: Soluble fms-like tyrosine kinase 1
SHAP: Shapley additive explanations
STING: Stimulator of interferon genes
SVM: Support vector machine
UTR: Untranslated region
VEGF: Vascular endothelial growth factor
XGB: Extreme gradient boosting
