# Supplementary material for: Discovery and validation of GNA12circle as a first-trimester plasma eccDNA marker for early-onset preeclampsia
Source: Front Neurol. 2026 Aug 25;17:1900036. doi: 10.3389/fneur.2026.1900036 (PMC13549907; doi:10.3389/fneur.2026.1900036)
Supplement: Supplementary file 1 [file Image_1.PDF]

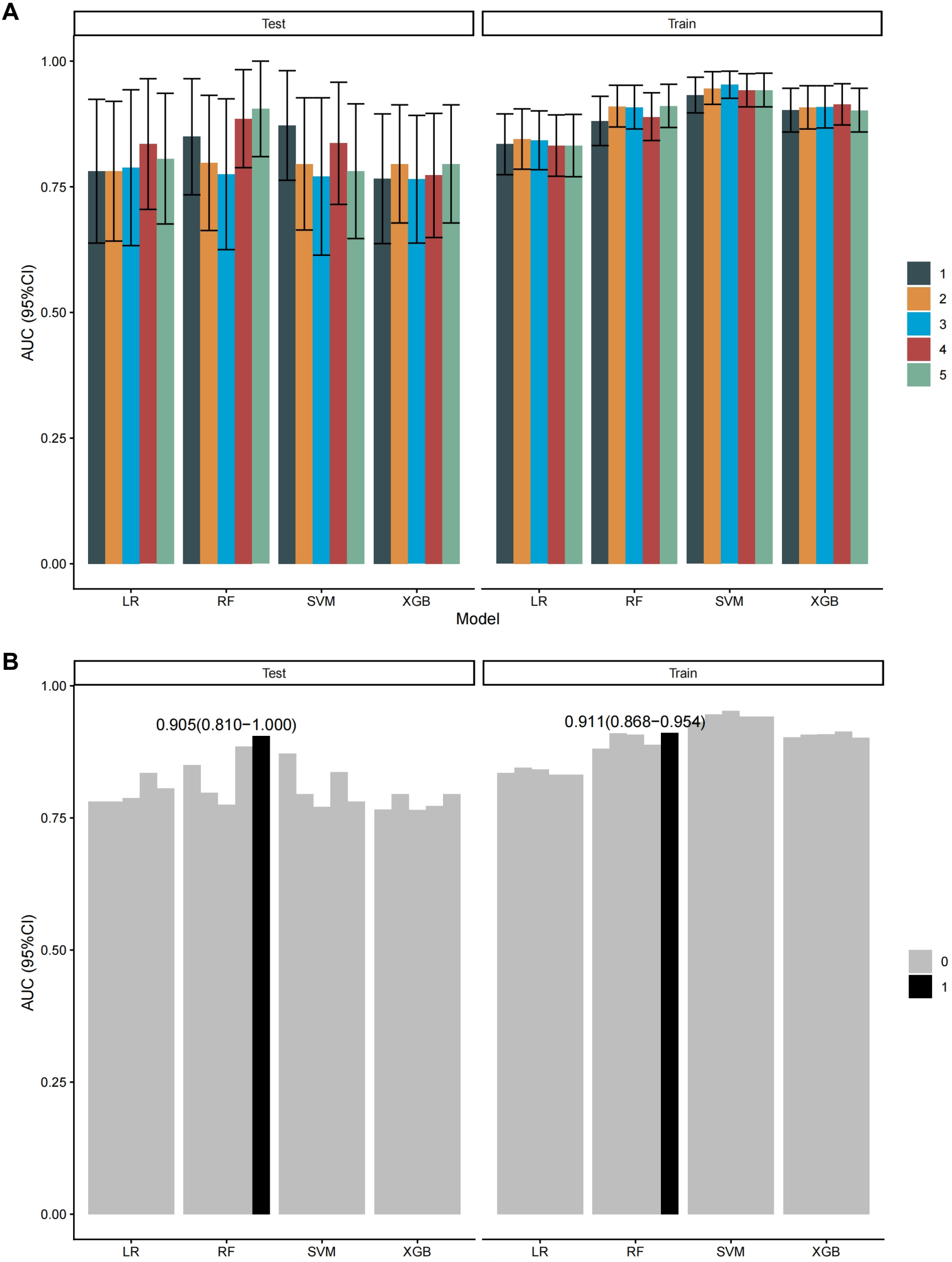
A
Test
Train
1.00
0.75
0.50
0.25
0.00
AUC (95%CI)
LR
RF
SVM
XGB
Model
1
2
3
4
5
B
Test
Train
0.905(0.810−1.000)
0.911(0.868−0.954)
1.00
0.75
0.50
0.25
0.00
AUC (95%CI)
LR
RF
SVM
XGB
0
1
