# Supplementary material for: Discovery and validation of GNA12circle as a first-trimester plasma eccDNA marker for early-onset preeclampsia
Source: Front Neurol. 2026 Aug 25;17:1900036. doi: 10.3389/fneur.2026.1900036 (PMC13549907; doi:10.3389/fneur.2026.1900036)
Supplement: Supplementary file 2 [file Table_1.DOCX]

Table S1. Prioritization metrics of increased- and decreased-abundance candidate eccDNAs from the exploratory discovery analysis.

| **Direction in EOPE** | **Direction-specific rank** | **Genomic coordinates** | **Gene** | **Biotype** | **Length (bp)** | **logFC** | **Nominal P value** | **FDR** | **Detection in EOPE** | **Detection in controls** | **Mean raw abundance EOPE** | **Mean raw abundance controls** | **Key annotation/rationale** |
| --- | --- | --- | --- | --- | --- | --- | --- | --- | --- | --- | --- | --- | --- |
| **Increased**  **in EOPE** | 1 | chr15:67359388-67359724 | SMAD3 | protein coding | 336 | 7.875 | 0.0128 | 0.146 | 3/5 | 0/5 | 14.40 | 0.00 | FoxO signaling pathway; Cell cycle; Endocytosis; Wnt signaling pathway; TGF-beta signaling pathway; Wnt signaling pathway; TGF-beta signaling pathway; Hippo signaling pathway; positive regulation of focal adhesion assembly; negative regulation of transcription from RNA polymerase II promoter |
|  | 2 | chr7:2876332-2876692 | GNA12 | protein coding | 360 | 6.806 | 0.0250 | 0.146 | 3/5 | 0/5 | 5.80 | 0.00 | MAPK signaling pathway; cGMP-PKG signaling pathway; Sphingolipid signaling pathway; MAPK signaling pathway; cGMP-PKG signaling pathway; platelet activation; Rho protein signal transduction; regulation of fibroblast migration |
|  | 3 | chr3:138551800-138552033 | PIK3CB | protein coding | 233 | 5.331 | 0.0332 | 0.146 | 3/5 | 0/5 | 2.80 | 0.00 | Inositol phosphate metabolism; ErbB signaling pathway; Ras signaling pathway; Rap1 signaling pathway; cGMP-PKG signaling pathway; Ras signaling pathway; Rap1 signaling pathway; cGMP-PKG signaling pathway; cAMP signaling pathway; HIF-1 signaling pathway |
|  | 4 | chr12:2547777-2547968 | CACNA1C | protein coding | 191 | 6.398 | 0.0242 | 0.146 | 4/5 | 0/5 | 3.80 | 0.00 | MAPK signaling pathway; Calcium signaling pathway; cGMP-PKG signaling pathway; cAMP signaling pathway; Cardiac muscle contraction; MAPK signaling pathway; Calcium signaling pathway; cGMP-PKG signaling pathway; cAMP signaling pathway; Vascular smooth muscle contraction |
|  | 5 | chr19:54389300-54389651 | PRKCG | protein coding | 351 | 7.786 | 0.0131 | 0.146 | 3/5 | 0/5 | 15.20 | 0.00 | MAPK signaling pathway; ErbB signaling pathway; Ras signaling pathway; Rap1 signaling pathway; Calcium signaling pathway; MAPK signaling pathway; Ras signaling pathway; Rap1 signaling pathway; Calcium signaling pathway; HIF-1 signaling pathway |
|  | 6 | chr1:178400767-178400943 | RASAL2 | protein coding | 176 | 6.808 | 0.0250 | 0.146 | 3/5 | 0/5 | 5.00 | 0.00 | Ras signaling pathway; Ras signaling pathway; MAPK cascade; negative regulation of Ras protein signal transduction; Ras protein signal transduction; activation of MAPKK activity |
|  | 7 | chr16:1815797-1816032 | MAPK8IP3 | protein coding | 235 | 6.259 | 0.0322 | 0.146 | 3/5 | 0/5 | 6.00 | 0.00 | MAPK signaling pathway; in utero embryonic development |
|  | 8 | chr11:48178109-48178277 | PTPRJ | protein coding | 168 | 6.020 | 0.0351 | 0.146 | 3/5 | 0/5 | 4.00 | 0.00 | Adherens junction; negative regulation of vascular permeability; platelet-derived growth factor receptor signaling pathway; regulation of cell adhesion; negative regulation of cell migration; negative regulation of platelet-derived growth factor receptor signaling pathway |
|  | 9 | chr9:136817931-136818115 | VAV2 | protein coding | 184 | 3.769 | 0.0253 | 0.146 | 3/5 | 0/5 | 0.80 | 0.00 | Rap1 signaling pathway; cAMP signaling pathway; Chemokine signaling pathway; Focal adhesion; Natural killer cell mediated cytotoxicity; Rap1 signaling pathway; cAMP signaling pathway; Focal adhesion; Leukocyte transendothelial migration; Regulation of actin cytoskeleton |
|  | 10 | chr10:97267648-97267890 | SORBS1 | protein coding | 242 | 7.485 | 0.0161 | 0.146 | 5/5 | 0/5 | 9.60 | 0.00 | PPAR signaling pathway; Adherens junction; Insulin signaling pathway; focal adhesion assembly; cell-matrix adhesion; focal adhesion |
| **Decreased in EOPE** | 1 | chr18:807577-807768 | YES1 | protein coding | 191 | -8.568 | 0.0184 | 0.146 | 0/5 | 3/5 | 0.00 | 43.80 | Adherens/tight junction pathways and platelet-derived growth factor response; relevant to cell–cell junction, vascular signaling, and endothelial/placental dysfunction. |
|  | 2 | chr6:6193090-6193281 | F13A1 | protein coding | 191 | -8.568 | 0.0184 | 0.146 | 0/5 | 3/5 | 0.00 | 44.00 | Complement/coagulation cascade, platelet activation, and blood coagulation; relevant to PE-associated coagulation and placental vascular pathology. |
|  | 3 | chr8:56901761-56902077 | LYN | protein coding | 316 | -7.854 | 0.0260 | 0.146 | 0/5 | 3/5 | 0.00 | 26.80 | Chemokine and NF-kappa B signaling; immune/inflammatory signaling and p53-related annotation. |
|  | 4 | chr19:17935906-17936241 | JAK3 | protein coding | 335 | -7.287 | 0.0347 | 0.146 | 0/5 | 3/5 | 0.00 | 32.80 | Chemokine and PI3K-Akt signaling with T-cell activation annotations; relevant to immune/inflammatory and signaling mechanisms. |
|  | 5 | chr7:92462741-92463070 | CDK6 | protein coding | 329 | -7.030 | 0.0405 | 0.146 | 0/5 | 3/5 | 0.00 | 36.20 | Cell cycle, p53 signaling, and PI3K-Akt signaling; relevant to proliferation/stress pathways in placental dysfunction. |
|  | 6 | chr3:10387219-10387557 | ATP2B2 | protein coding | 338 | -7.779 | 0.0268 | 0.146 | 0/5 | 4/5 | 0.00 | 34.00 | Calcium signaling and cGMP-PKG signaling; 4/5 controls detected, suitable fragment length. |
|  | 7 | chr1:213131484-213131826 | VASH2 | protein coding | 342 | -7.988 | 0.0245 | 0.146 | 0/5 | 3/5 | 0.00 | 28.80 | Positive regulation of endothelial cell proliferation; direct endothelial/angiogenesis-related annotation. |
|  | 8 | chr1:243870050-243870237 | AKT3 | protein coding | 187 | -7.049 | 0.0404 | 0.146 | 0/5 | 3/5 | 0.00 | 15.00 | MAPK/ErbB/Ras-related signaling and kinase activity; relevant to major intracellular signaling pathways. |
|  | 9 | chr10:105408195-105408407 | SH3PXD2A | protein coding | 212 | -6.957 | 0.0424 | 0.146 | 0/5 | 3/5 | 0.00 | 14.40 | Podosome-related annotation and superoxide metabolic process; relevant to migration/invasion and oxidative stress contexts. |
|  | 10 | chr7:14246982-14247173 | DGKB | protein coding | 191 | -8.257 | 0.0215 | 0.146 | 0/5 | 3/5 | 0.00 | 35.40 | Platelet activation, calcium ion binding, and glycerolipid/glycerophospholipid metabolism annotations. |

Note: Candidate eccDNAs were prioritized from the exploratory discovery analysis using nominal differential abundance, detection pattern across discovery samples, fragment length suitable for junction-specific validation, technical feasibility for primer design, and biological plausibility. The table includes the top 10 increased-abundance and top 10 decreased-abundance candidate eccDNAs prioritized under the same framework. FDR values were calculated to account for multiple testing; however, none of the listed candidates reached FDR <0.05. Therefore, these eccDNAs should be interpreted as nominally differentially abundant exploratory candidates rather than genome-wide significant findings. Detection frequency is shown as the number of samples with detectable raw counts in each group. EOPE, early-onset preeclampsia; eccDNA, extrachromosomal circular DNA; FDR, false discovery rate; logFC, log2 fold change.
