# Supplementary material for: Discovery and validation of GNA12circle as a first-trimester plasma eccDNA marker for early-onset preeclampsia
Source: Front Neurol. 2026 Aug 25;17:1900036. doi: 10.3389/fneur.2026.1900036 (PMC13549907; doi:10.3389/fneur.2026.1900036)
Supplement: Supplementary file 3 [file Table_2.DOCX]

Table S2 Primers in this study.

| **Group** | **Gene** | **Sequence** |
| --- | --- | --- |
| outward PCR | GNA12^circle^ | F: ACATCAGCAGAGAAACCACA |
|  |  | R: CCAGGTTCAAGCGATTCTCC |
| qPCR | GNA12^circle^ | F: ACATCAGCAGAGAAACCACA |
|  |  | R: CCAGGTTCAAGCGATTCTCC |
|  | pGEX-5X-2 | F: GGGCTGGCAAGCCACGTTTGGTG |
|  |  | R: CCGGGAGCTGCATGTGTCAGAGG |

F: Forward, R: Reverse, GNA12^circle^: eccDNA GNA12^circle^ at Chr7:2876332–2876692.
