# Supplementary material for: Discovery and validation of GNA12circle as a first-trimester plasma eccDNA marker for early-onset preeclampsia
Source: Front Neurol. 2026 Aug 25;17:1900036. doi: 10.3389/fneur.2026.1900036 (PMC13549907; doi:10.3389/fneur.2026.1900036)
Supplement: Supplementary file 4 [file Table_3.DOCX]

Table S3. Quality-control metrics for the qPCR assays

| **QC metric** | **Result** |
| --- | --- |
| Biological samples | 218 |
| Technical replicates per sample | 3 |
| GNA12^circle^ Ct, median (IQR) | 25.29 (24.48–27.74) |
| Spike-in Ct, median (IQR) | 16.45 (16.32–16.58) |
| GNA12^circle^ triplicate Ct SD, median (IQR) | 0.111 (0.069–0.188) |
| Spike-in triplicate Ct SD, median (IQR) | 0.076 (0.046–0.116) |
| Triplicates with Ct SD ≤0.5 cycles | 100.00% |
| GNA12^circle^ intra-assay CV, median (IQR) | 7.74% (4.80%–13.04%) |
| Spike-in intra-assay CV, median (IQR) | 5.27% (3.15%–8.11%) |
| Standard calibrator inter-assay CV after spike-in normalization | 6.19% |

CVs were calculated from 2^-Ct^-transformed relative quantity values. Spike-in-normalized inter-assay CV was calculated from 2^-ΔCt^ values, where ΔCt = Ct(GNA12circle) − Ct(spike-in). Standard calibrator wells were excluded from study-sample Ct distribution analyses.
