# Supplementary material for: Discovery and validation of GNA12circle as a first-trimester plasma eccDNA marker for early-onset preeclampsia
Source: Front Neurol. 2026 Aug 25;17:1900036. doi: 10.3389/fneur.2026.1900036 (PMC13549907; doi:10.3389/fneur.2026.1900036)
Supplement: Supplementary file 5 [file Table_4.DOCX]

Table S4 Quality control of Circle-seq.

| **Sample** | **Raw Reads** | **Clean Reads** | **Aligned Reads** | **Aligned Rate** | **Q30** |
| --- | --- | --- | --- | --- | --- |
| EOPE 1 | 319,859,276 | 319,841,016 | 289,874,166 | 90.63% | 90.67% |
| EOPE 2 | 288,376,440 | 288,359,048 | 274,118,136 | 95.06% | 90.58% |
| EOPE 3 | 300,545,300 | 300,528,420 | 281,039,194 | 93.52% | 88.44% |
| EOPE 4 | 360,337,416 | 360,316,920 | 354,466,910 | 98.38% | 89.63% |
| EOPE 5 | 291,543,592 | 291,526,704 | 266,548,294 | 91.43% | 88.64% |
| Control 1 | 268,147,616 | 268,128,220 | 227,373,984 | 84.80% | 84.75% |
| Control 2 | 279,091,608 | 279,070,736 | 237,168,190 | 84.98% | 88.30% |
| Control 3 | 258,831,588 | 258,809,732 | 211,090,782 | 81.56% | 88.39% |
| Control 4 | 311,027,664 | 311,002,932 | 272,660,456 | 87.67% | 88.25% |
| Control 5 | 249,897,556 | 249,879,692 | 225,342,350 | 90.18% | 89.20% |

EOPE: Early-onset preeclampsia.
