# Supplementary material for: Discovery and validation of GNA12circle as a first-trimester plasma eccDNA marker for early-onset preeclampsia
Source: Front Neurol. 2026 Aug 25;17:1900036. doi: 10.3389/fneur.2026.1900036 (PMC13549907; doi:10.3389/fneur.2026.1900036)
Supplement: Supplementary file 6 [file Table_5.DOCX]

Supplementary Table S5. Comparison of the combined RF model and clinical-only RF model across cross-validation folds.

|  |  | **Fold** | **AUC(95%CI)** | **accuracy(95% CI)** | **sensitivity(95%CI)** | **Specificity(95%CI)** | **PPV(95%CI)** | **NPV(95%CI)** | **Cut off** | **Delong test-P value** |
| --- | --- | --- | --- | --- | --- | --- | --- | --- | --- | --- |
| Combined RF model | Train | 5 | 0.911(0.868-0.954) | 0.828(0.763-0.881) | 0.759(0.669-0.849) | 0.897(0.833-0.961) | 0.880(0.806-0.954) | 0.788(0.707-0.868) | 0.675 |  |
| Combined RF model | Test | 5 | 0.905(0.810-1.000) | 0.795(0.647-0.902) | 0.864(0.720-1.000) | 0.727(0.541-0.913) | 0.760(0.593-0.927) | 0.842(0.678-1.000) |  |  |
| Clinical-only RF model | Train | 1 | 0.767(0.697-0.837) | 0.709(0.635-0.775) | 0.816(0.735-0.897) | 0.602(0.500-0.705) | 0.670(0.580-0.759) | 0.768(0.669-0.868) | 0.525 | 0.000653[1] |
| Clinical-only RF model | Test | 1 | 0.709(0.548-0.870) | 0.651(0.491-0.790) | 0.818(0.657-0.979) | 0.476(0.263-0.690) | 0.621(0.444-0.797) | 0.714(0.478-0.951) |  | 0.04351[2] |
| Clinical-only RF model | Train | 2 | 0.786(0.719-0.853) | 0.718(0.645-0.784) | 0.701(0.605-0.797) | 0.736(0.643-0.828) | 0.726(0.631-0.822) | 0.711(0.617-0.805) | 0.425 | 0.00213[1] |
| Clinical-only RF model | Test | 2 | 0.564(0.388-0.740) | 0.591(0.432-0.737) | 0.636(0.435-0.837) | 0.545(0.337-0.754) | 0.583(0.386-0.781) | 0.600(0.385-0.815) |  | 0.0007137[2] |
| Clinical-only RF model | Train | 3 | 0.770(0.703-0.838) | 0.691(0.617-0.759) | 0.807(0.724-0.889) | 0.575(0.471-0.679) | 0.657(0.568-0.747) | 0.746(0.642-0.850) | 0.525 | 0.0006394[1] |
| Clinical-only RF model | Test | 3 | 0.660(0.493-0.828) | 0.558(0.399-0.709) | 0.714(0.521-0.908) | 0.409(0.204-0.615) | 0.536(0.351-0.720) | 0.600(0.352-0.848) |  | 0.01512[2] |
| Clinical-only RF model | Train | 4 | 0.805(0.742-0.869) | 0.736(0.664-0.799) | 0.759(0.669-0.849) | 0.713(0.618-0.808) | 0.725(0.634-0.817) | 0.747(0.653-0.841) | 0.425 | 0.006342[1] |
| Clinical-only RF model | Test | 4 | 0.646(0.481-0.810) | 0.545(0.388-0.696) | 0.545(0.337-0.754) | 0.545(0.337-0.754) | 0.545(0.337-0.754) | 0.545(0.337-0.754) |  | 0.00779[2] |
| Clinical-only RF model | Train | 5 | 0.773(0.705-0.841) | 0.718(0.645-0.784) | 0.759(0.669-0.849) | 0.678(0.580-0.776) | 0.702(0.610-0.795) | 0.737(0.641-0.834) | 0.575 | 0.0003207[1] |
| Clinical-only RF model | Test | 5 | 0.616(0.446-0.786) | 0.568(0.410-0.717) | 0.636(0.435-0.837) | 0.500(0.291-0.709) | 0.560(0.365-0.755) | 0.579(0.357-0.801) |  | 0.002457[2] |

The combined RF model included GNA12^circle^ and routine first-trimester variables. The clinical-only RF model included the same routine first-trimester variables but excluded GNA12^circle^. P values were calculated using paired DeLong tests to compare AUCs between the two models within the corresponding training folds and held-out test folds. P < 0.05 was considered statistically significant. [1] P value for comparison between the combined RF model and clinical-only RF model in the training fold. [2] P value for comparison between the two models in the held-out test fold.
