# Supplementary material for: Discovery and validation of GNA12circle as a first-trimester plasma eccDNA marker for early-onset preeclampsia
Source: Front Neurol. 2026 Aug 25;17:1900036. doi: 10.3389/fneur.2026.1900036 (PMC13549907; doi:10.3389/fneur.2026.1900036)
Supplement: Supplementary file 7 [file Table_6.DOCX]

Table S6. Prevalence-adjusted positive and negative predictive value estimates of the random forest model under assumed early-onset preeclampsia prevalences.

| **Assumed EOPE prevalence** | **Sensitivity** | **Specificity** | **Adjusted PPV** | **Adjusted NPV** |
| --- | --- | --- | --- | --- |
| 0.5% | 0.706 | 0.788 | 1.6% | 99.8% |
| 1.0% | 0.706 | 0.788 | 3.2% | 99.6% |

PPV and NPV were recalculated using the cross-validated mean sensitivity and specificity of the random forest model under assumed EOPE prevalences of 0.5% and 1.0%. These estimates were intended to illustrate the potential impact of disease prevalence on predictive values and assume that sensitivity and specificity remain stable across populations.
